# Synovial Sarcoma- A Rare Tumor of the Larynx

**Published:** 2016-05

**Authors:** Ghodrat Mohammadi, Arezu khansarinia

**Affiliations:** 1 *Department of Otorhinolaryngology, Tabriz university of Medical Science, Tabriz, Iran.*

**Keywords:** Synovial sarcoma, Larynx, Malignant mesenchymal tumors

## Abstract

**Introduction:**

Malignant mesenchymal tumors of the larynx are rare. One type of malignant mesenchymal tumor is synovial sarcoma with unknown histogenesis, which occurs predominantly in the lower extremities of young adults. The head and neck region is a relatively rare location. There are few cases of malignant mesenchymal tumors with laryngeal localization in literature.

**Case Report:**

In this report, a new case in a 23-year-old man, which was referred with increasing hoarseness for eight months, and dysphagia, odynophagia, and dyspnea since nearly one year ago, is reported. Indirect laryngoscopy revealed a laryngeal submucosal mass. The patient was operated and the histopathological diagnosis of synovial sarcoma was confirmed by IHC (Immunohistochemisry).

**Conclusion:**

Synovial sarcoma occurs predominantly in the lower extremities of young adults. Because very few cases of laryngeal synovial sarcoma are reported, every new case will bring some new information about diagnosis and therapy. It is of utmost importance to get to know new aspects and therapeutical modalities of this rare tumor.

## Introduction

Synovial sarcomas are mesenchymal malignancies that most often affect the lower extremities of young adults. These tumors are classified into monophasic and biphasic variants. IHC plays a major role in diagnosis (1). About 3- 9% of cases were reported in the head and neck region while laryngeal involvement is rare, which makes the correct diagnosis difficult for the pathologist and the clinician (2,3). The Treatment of synovial sarcoma is surgery, radiation therapy, and chemotherapy (4). About 18 cases of synovial sarcoma of the larynx were reported in the English-language literature (5). In this case report, a 23-year-old man, who presented with odynophagia, dysphagia, and voice change, is reported. To the best of our knowledge, this is another case of synovial sarcoma of the larynx.

## Case Report

**Fig 1:**
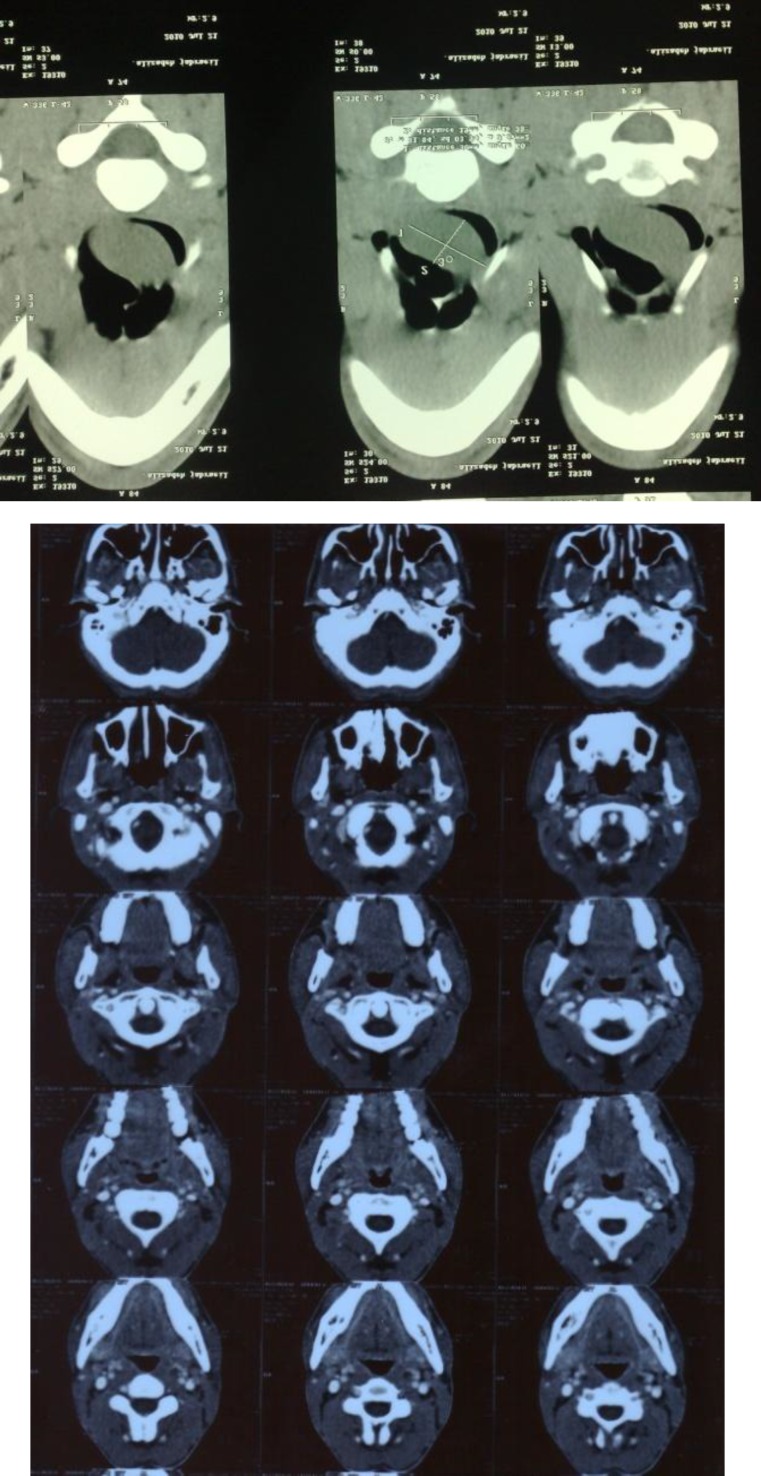
Computed tomography scan shows a solid mass, which extended from the hypo pharynx into the epiglottis and aryepiglottis fold

A 23-year-old male was admitted to the ENT department of the University Hospital because of increasing hoarseness for eight months, and dysphagia, odynophagia, and dyspnea since nearly one year ago. He had no weight loss, otalgia, hemoptysis, or constitutional signs of any diseases. Upon indirect laryngoscopic examination, an endolaryngeal submucosal mass, involving the epiglottis and aryepiglottis fold, was discovered. True vocal cords were normal. No masses were palpable in his neck. The results of laboratory studies were normal. A computed tomography (CT) scan was valuable in determining the site of origin and extent of the lesion, that revealed a solid mass, which extended from the hypopharynx into the epiglottis and the area of the aryepiglottis fold, particularly in the left side (Fig.1). Using the microlaryngoscopic procedure, a biopsy sampling was taken from the laryngeal mass. Following that, a wide surgical excision of the tumor with a free margin was performed. Radiotherapy was added.

***Pathology***

**Fig 2:**
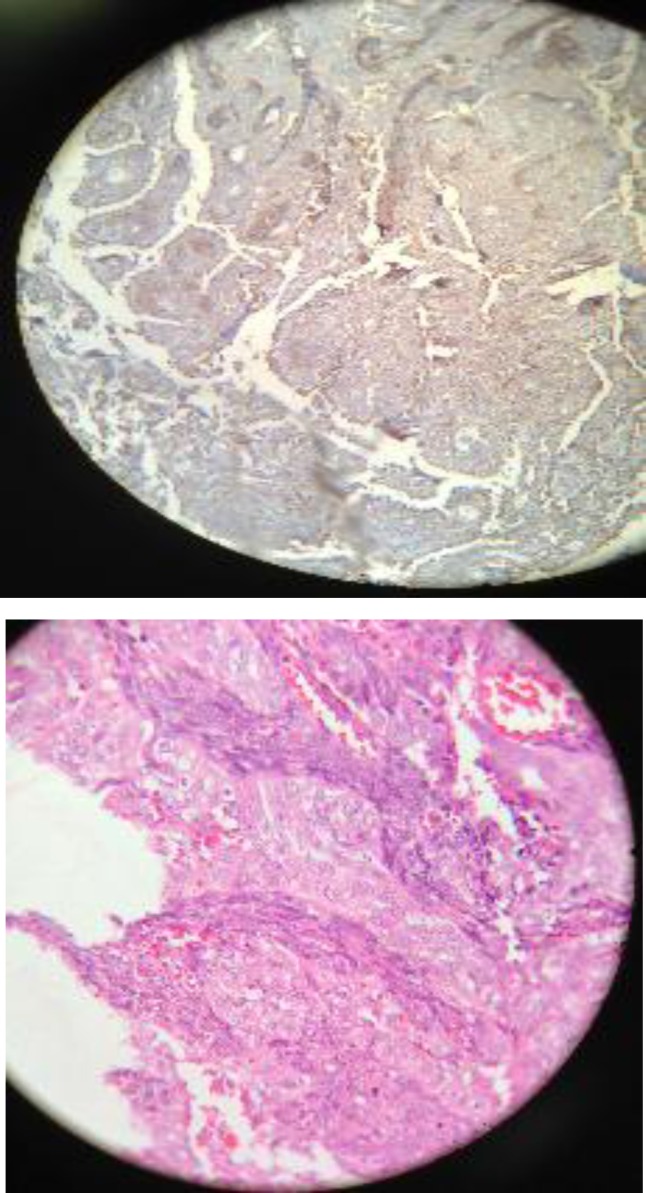
Well differentiated synovial sarcoma of the biphasic type with left supraglottic involvement

Microscopic examination reveals fragments of tissue including a neoplasm. It is composed of proliferated epithelial like cellular elements arranged in aggregated and gland like structures with intervening proliferated fibroblastic spindle cells. The neoplasm, found superficially, is papillomatous and forming a separate pseudopapillary vegetation. The epithelial component is more abundant in areas where the neoplastic cells show some atypia and atypical mitotic figures. Located in the deepest portion are spindle cells, which are prominent and show a noticeable pleomorphism and mitotic index in the most active area. IHC stains are positive for CD99 in both spindle and epithelial cells, positive for CK only in epithelial cells, positive for EMA in epithelial cells, weakly positive for EMA in spindle cells, and negative for BCI2. Diagnosis was well differentiated synovial sarcoma of the biphasic type with left supraglottic involvement (Fig.2). 

IHC stains are positive for CD99 in both spindle and epithelial cells, positive for CK only epithelial cells, positive for EMA in epithelial, weakly positive for EMA in spindle cells, and negative for BCI 2.

***Treatment***

A wide surgical excision of the tumor was planned. The tumor extended from the hypopharynx into the epiglottis and the area of the aryepiglottis fold in the left side and was excised with free margins. 

Elective radical neck dissection was not performed; but the patient received radiotherapy after surgery with no recurrence after 42 months.

## Discussion

Synovial sarcoma of the larynx was first described early in the 20th century. The least frequent site of occurrence is the larynx. Until 1975, only four cases have been reported (6). Since then, only 19 cases have been reported, including the current case (Table.1) (6). The reported cases included 14 males, 3 females, and one case in which the patient’s sex was not reported (5). Hoarseness, upper respiratory distress, and dysphagia characterize the original complaints in laryngopharyngeal synovial sarcoma (6). Our case presented with increasing hoarseness for eight months, and dysphagia, odynophagia, and dyspnea since nearly one year ago. He had no weight loss, otalgia, and hemoptysis. In our case, true vocal cords were normal and the mass was found submucosally in the supraglotic region and hypopharynx.

Primary tumors of the larynx are predominantly classified as squamous cell carcinoma. Other unusual tumors in this location are fibrosarcoma, chondrosarcoma, osteosarcoma, and rhabdomyosarcoma (7,8).

Biphasic synovial sarcoma causes very few problems in its diagnosis; however, synovial sarcoma can display different patterns. The monophasic fibrous type can be misdiagnosed as a spindle cell sarcoma or a hemangiopericytoma (9). Diagnosis of our cases after IHC stains showed well differentiated synovial sarcoma of the biphasic type. The optimal treatment strategy for synovial sarcoma has not yet been established. Although surgical excision with a wide margin is currently performed as the first treatment of choice, the efficacy of other treatments such as chemotherapy or radiotherapy has not yet been debated (10). Lymph node metastasis is rare and hematogenous spread is more probable, with the lung being the most common site of metastasis. Routine radical neck dissection is not recommended. In this case report, we present a case of synovial sarcoma of the larynx, which was treated with a wide surgical excision with a safe margin, after which the patient received radiotherapy with no recurrence after 42 months.

## Conclusion

With this case report, another new case of synovial sarcoma of the endolarynx is added to literature. Due to the presence of very few cases of laryngeal synovial sarcomas, every new case will bring some new information about diagnosis and therapy. Every case of synovial sarcoma should be published because it is of utmost importance to get to know new aspects and therapeutical modalities of this rare tumor.
